# Sequencing the whole genome of a *Brucella abortus* using nanopore technology

**DOI:** 10.1128/mra.00366-25

**Published:** 2025-12-29

**Authors:** Munkhgerel Dalantai, Munkhjargal Tserendorj, Ulziisaikhan Gombosuren, Batbaatar Vanaabaatar, Myagmarsuren Punsantsogvoo, Battsetseg Badgar, Batsukh Zayat, Davaasuren Batdorj

**Affiliations:** 1 Laboratory of Molecular Genetics, Institute of Veterinary Medicine, Mongolian University of Life Sciences, Ulaanbaatar, Mongolia; 2 Department of Biology and Chemistry, Changwon National University 34925, Changwon, South Korea; 3 Laboratory of Helminthology, Institute of Veterinary Medicine, Mongolian University of Life, Ulaanbaatar, Mongolia; 4 Laboratory of Infection disease and Immunology, Institute of Veterinary Medicine, Mongolian University of Life, Ulaanbaatar, Mongolia; Loyola University Chicago, Chicago, Illinois, USA

**Keywords:** *Brucella abortus*, MinION, Y11_MNG, Oxford Nanopore technology

## Abstract

We report the whole-genome sequence of the *B. abortus* strain “Y11_MNG” isolated from Mongolia. The MinION platform was used for sequencing and draft assembly composed of six contigs with a length of 3,274,836 bp. Assembly completeness was 93.5%, with 116 of 124 single-copy genes present.

## ANNOUNCEMENT

Brucellosis is a widespread zoonotic disease caused by *Brucella*—nonmotile, gram-negative coccobacilli (1). Among its subspecies, *Brucella abortus* mainly infects bovines, as well as elk and camels (2). The genus includes six species categorized by their primary hosts. Livestock-related species include *B. melitensis* (sheep/goats), *B. abortus* (cattle), *B. suis* (pigs), and *B. ovis* (sheep) (3). Due to sanitation, social, and political factors and global travel, human brucellosis epidemiology has shifted in recent years (4). Since the 1940s, it has burdened Mongolia’s public health and economy (5), with *Brucella* widespread in local livestock (6). In the last 30 years, over 1,000 *B. abortus* genomes have been published—only two from yaks (7).

This study sequenced the whole genome of *Brucella abortus*. The strain “Y11_MNG” was isolated in 2014 from a swab taken from the uterus of an aborted yak cow (*Bos grunniens*) in Arkhangai Province, Mongolia. This study was approved by the Ethics Committee of the Institute of Veterinary Medicine in Mongolia (Approval No.23/01/07). Our findings provide further insight into its pathogenicity and genetic profile.

For sample preparation, a single colony of the Y11_MNG strain was cultured on CITA medium prepared with Blood Agar Base No. 2 (Oxoid Code SR083) with antibiotics (vancomycin, colistin, nystatin, nitrofurantoin), 5% calf serum (Invitrogen) at 37°C under 5% CO_₂_-enriched conditions for up to 48 h.

Genomic DNA was extracted using the PCI method (25:24:1, v/v) (8). DNA quantity and quality were assessed using a Qubit 3.0 fluorometer (Thermo Fisher Scientific). The sequencing library was prepared following the SQK-PBK004 protocol with the PCR barcoding kit and reagents, including NEBNext FFPE Repair Mix and NEBNext Ultra II End Repair/dA-tailing Module. DNA was sequenced using the MinION with an R9.4.1 flow cell following the EXP-FLP002 protocol without shearing and size selection. Base calling and quality control were performed with MinKNOW v19.06.8. Adapters and lambda control DNA were removed by using Porechop v0.2.4 (9) and Nanolyse v1.2.1 (10). Whole-genome assembly was performed using Flye v2.81 (11) with parameter settings: estimated genome size (-g 3.3m) and number of threads (-t 16). Flye results were refined and reconciled using Trycycler v0.5.5 (12).

The genome statistics were calculated using SeqKit v2.3.1 (13). The final assembly quality and completeness were assessed using QUAST v5.2.0 (14) and BUSCO v5.6.1 (15). The genome assembly was annotated using the NCBI Prokaryotic Genome Annotation Pipeline (PGAP) v6.5 (16). All bioinformatics tools in this study were run using default parameters, unless specified.

Sequencing with the MinION platform generated 116,630 reads (read N50 = 2,317 bp). The genome assembly was generated from these reads, yielding six contigs with a total length of 3,240,341 bp, a GC content of 57.2%, and a mean coverage of 60.5× (N50 = 964,736 bp; largest contig, 1,379,900 bp). The assembly exhibited 93.5% completeness according to BUSCO analysis, with 116 of 124 genes detected. Among the genes, 115 genes are single-copy, and one gene (Methyltransf_5 MraW methylase) is duplicated. An average nucleotide identity analysis (pyANI v0.3.0 alpha) showed 99.87% identity with *Brucella abortus* S19 (NC_010742).

The complete genomic sequence of *B. abortus* provides an essential resource for investigating pathogenicity and virulence factors.

## Data Availability

The complete genomic sequence has been deposited in DDBJ/ENA/GenBank under accession number JASTZV000000000, and the raw sequence reads are publicly available in the NCBI Sequence Read Archive (SRA) under accession numbers SRR24087961 to SRR24087991.
